# A phase II trial with rosiglitazone in liposarcoma patients

**DOI:** 10.1038/sj.bjc.6601306

**Published:** 2003-10-14

**Authors:** G Debrock, V Vanhentenrijk, R Sciot, M Debiec-Rychter, R Oyen, A Van Oosterom

**Affiliations:** 1 Department of Medical Oncology, University Hospital Gasthuisberg, Universiteit Leuven, Herestraat 49, Leuven 3000, Belgium; 2 Department of Pathology, University Hospital Gasthuisberg, Universiteit Leuven, Herestraat 49, Leuven 3000, Belgium; 3 Department of Human Genetics, University Hospital Gasthuisberg, Universiteit Leuven, Herestraat 49, Leuven 3000, Belgium; 4 Department of Radiology, University Hospital Gasthuisberg, Universiteit Leuven, Herestraat 49, Leuven 3000, Belgium

**Keywords:** liposarcoma, rosiglitazone, PPAR*γ*

## Abstract

Agents of the thiazolidinedione drug family can terminally differentiate human liposarcoma cells *in vitro* by activating genes responsible for lipocyte differentiation. One study has shown clinical activity of troglitazone treatment in liposarcoma patients. We sought to find further evidence for this result. In all, 12 patients with a liposarcoma received rosiglitazone 4 mg b.d. They were followed clinically and with repeated biopsies for histological and biological studies. At the molecular level the mRNA translation of three genes that are induced by this treatment (peroxisome proliferator-activated receptor *γ* (PPAR*γ*), adipsin and fatty acid binding protein) was determined. Nine patients were eligible for evaluation. One patient had to stop treatment due to hepatotoxicity. The mean time to progression was 6 months (2 – 16 months), with one patient still on treatment. We did not see any significant change in histologic appearance of the liposarcomas by the treatment. The level of gene expression changed significantly in two patients, but this did not result in a clinical response. Based on this study, rosiglitazone is not effective as an antitumoral drug in the treatment of liposarcomas. Increased PPAR*γ* activity does not correlate with the clinical evolution.

Soft-tissue sarcomas are a group of mesodermal tumours encompassing many histotypes. Liposarcomas have a peak incidence between age 50 and 65 years. Several subtypes exist, ranging from low- to high-grade malignancy (well-differentiated-, myxoid-, round cell-, pleomorphic-, mixed- and dedifferentiated-type liposarcomas). Large tumours can consist of more than one liposarcoma subtype, thus containing better types of differentiation (Meis-Kindblom *et al*, 2001). By genetic analysis, a characteristic t(12;16) translocation has been found for the myxoid/round cell subtypes, generating a TLS-CHOP fusion protein (Crozat *et al*, 1993; Knight *et al*, 1995). The exact function of this transcript remains to be elucidated. Several forms of this fusion protein have been described, but none of them seems to be of prognostic value. A minority of these liposarcomas show a different translocation, t(12;22), producing the EWS-CHOP fusion transcript (Antonescu *et al*, 2001). Well-differentiated or dedifferentiated liposarcomas are characterised by supernummery ring and/or giant marker chromosomes with amplified sequences of chromosomal region 12q (Dei Tos, 2000).

Being notoriously resistant to chemo- and radiotherapy, the major prognostic factors in the treatment of liposarcomas are their resectability and grade. Metastatic liposarcoma is hardly curable. However, the gain in knowledge about the molecular biology of the normal differentiation of lipocytes has opened new treatment perspectives. The peroxisome proliferator-activated receptor-*γ* (PPAR*γ*) is a nuclear receptor that has been identified as a key player in the terminal differentiation of the adipocyte lineage (Tontonoz *et al*, 1994a). It forms a heterodimeric complex with the retinoid X receptor *α* that binds to specific DNA recognition sites and regulates the transcription of adipocyte-specific genes (Tontonoz *et al*, 1995). Ectopic expression and activation of PPAR*γ* in human fibroblasts induces a complete adipocytic phenotype (Tontonoz *et al*, 1994b). Several ligands for PPAR*γ* have been identified, both natural and synthetic (Vamecq and Latruffe, 1999). The thiazolidinedione class of drugs, which are oral antidiabetics, has been shown to be agonist ligands for PPAR*γ* (Lehmann *et al*, 1995). Treatment of liposarcoma cells in culture with pioglitazone has induced terminal differentiation of these cells (Tontonoz *et al*, 1997). In 1999, a clinical study with troglitazone showed evidence for differentiation of liposarcomas *in vivo* in three patients, assessing histological, biochemical, radiological and biological parameters (Demetri *et al*, 1999).

Based on these data, we followed nine liposarcoma patients in a phase II trial with rosiglitazone and documented clinical, histological and biological tumour parameters during the treatment.

## PATIENTS AND METHODS

### Patients and trial design

To be included in the study, patients had to have histologically confirmed liposarcoma; advanced (unresectable) disease that was measurable; prior surgery, chemotherapy or radiotherapy was allowed; a WHO performance status of 0, 1 or 2 and a life expectancy of at least 3 months. After obtaining an informed consent, all patients started a treatment with rosiglitazone at a dose of 4 mg b.d. Patients were seen at the outpatient department at baseline, at 6, 12, 20 and 32 weeks and at 1 year. CT scan imaging was performed at the same intervals to follow the evolution of the disease. Progressive disease was defined as a tumour volume increase of 100%, instead of the classical WHO criterion of 50%. This large cutoff was allowed because *in vitro* redifferentiated liposarcoma tissue has an increased cellular volume due to the intracellular accumulation of lipid (Tontonoz *et al*, 1997). Tumour sampling by CT- or ultrasound-guided needle biopsy (with a spring-driven device, supplied with a 14-gauge biopsy needle yielding biopsies of 1.9 cm in length) was also performed at the same intervals except at 20 weeks.

### Tumour tissue analysis

Biopsies were obtained for routine histologic staining with haematoxylin and eosin, molecular analysis and cytogenetics. Conventional classification of the histologic subtype was performed by a single sarcoma specialist pathologist (RS). Staining for PPAR*γ* was not routinely performed because no reliable antibody was at hand. Molecular analysis to quantify mRNA levels was performed by Taqman technology, following the manufacturer's instructions. This technique consists of two steps, namely an RT – PCR and the annealing of a fluorescently labelled probe between the two PCR primers. After processing, this probe yields a measurable signal and gives an exact figure that represents the level of a certain mRNA molecule in relation to an internal control molecule, glyceraldehyde-3-phosphate dehydrogenase (GADPH). The measured mRNA molecules included GADPH, PPAR*γ*, adipsin and FABP. The forward and reverse primers were 5′-agcctcaagatcatcagcaatg and 5′-atggactgtggtcatgagtcctt with the probe 5′-ccaactgcttagcacccctggcc for GADPH; 5′-accccctgttgtgttttcca and 5′-aatgggcttcacattcagcaa with the probe 5′-atgtgcttccccagaccgccc for PPAR*γ*; 5′-ccaagcgcctgtacgacgt and 5′-agcaggaggtcgtggtcgat with the probe 5′-ctccgcgcagtgccccacc for adipsin and 5′-tgtgcagaaatgggatggaaa and 5′-acgcattccaccaccagttt with the probe 5′-tcaaccaccataaagagaaaacgagagggatga for FABP.

Cytogenetic analysis was performed to identify t(12;16) translocations, typical for myxoid/round cell liposarcomas. Chromosome metaphases were obtained after short-term culture of a primary tumour sample, utilising standard procedures. G-banded chromosomes were evaluated and classified according to the International System of Human Cytogenetic Nomenclature (Mitelman, 1995, 37/id).

## RESULTS

In all, 12 patients were entered onto the study. Two patients became rapidly progressive and could not have a first control biopsy. One patient developed hepatotoxicity, which normalised after cessation of therapy, upon which the treatment was stopped.

Nine patients were eligible and evaluable for response. Their characteristics are presented in Table 1

**Table 1:** Patient characteristics

**Patient no. (age at diagnosis (years) – sex)**	**Histology**	**Translocation t(12;16)**	**Initial localisation**	**Pretreatment**	**Age at start treatment (years)**	**Duration of treatment**
1 (60 – F)	Myxoid	N	RP	Surgery/CT	72	6 months
2 (64 – M)	Myxoid/round cell	Y	Thigh	Surgery/CT	74	4 months
3 (55 – M)	Dedifferentiated	ND	RP	Surgery/CT	61	8 months
4 (61 – M)	Dedifferentiated	ND	RP	Surgery/CT	63	10 weeks
5 (53 – M)	Myxoid/dedifferentiated	N	RP	Surgery	55	2 months
6 (35 – F)	Myxoid/round cell	Y	Thigh	Surgery/RT	63	>16 months
7 (37 – M)	Myxoid/round cell	Y	RP	Surgery/RT	39	2 months
8 (46 – M)	Myxoid/round cell	N	Thigh	Surgery/CT/RT	48	5 months
9 (41 – F)	Well differentiated	ND	RP	Surgery/RT	46	9 months

Y=yes; N=no; ND=not determined; RP=retroperitonium; CT=chemotherapy; RT=radiotherapy

. The mean age at diagnosis of the liposarcoma and at the start of rosiglitazone treatment was 50 and 58 years, respectively. Six patients had myxoid/round cell liposarcomas and three of these had the t(12;16) translocation that characterises this subtype. The other histological subtypes were two dedifferentiated and one well-differentiated liposarcoma.

The median treatment duration was 5.5 months (2 – 16 months with one patient still on treatment), with at least one biopsy taken while on treatment.

**Figure 1:**
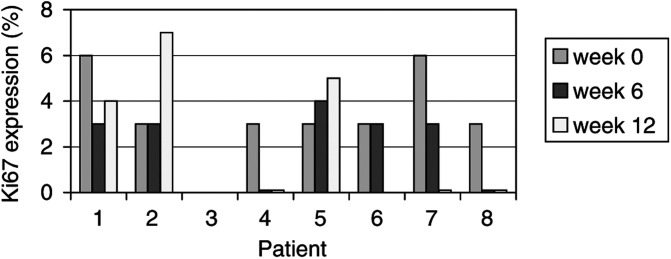
Ki-67 proliferation-associated antigen expression during treatment with rosiglitazone (data not available for patient 3)

The histopathological appearance of the tumour did not show any sign of redifferentiation by routine haematoxylin and eosin staining. An important variable was the problem of sampling error during the needle biopsies. As liposarcomas are often phenotypically heterogeneous, this can render reproducible sampling impossible. To check the cellular proliferation rates, expression of the Ki-67 nuclear antigen was assayed consecutively in tumour samples of eight patients (Figure 1

). The proliferation rate of the tumour cells was, as expected in these slowly growing tumours, very low at baseline. Although in more than half of the patients a decline was noted, it was not possible to detect any significant difference.

**Figure 2:**
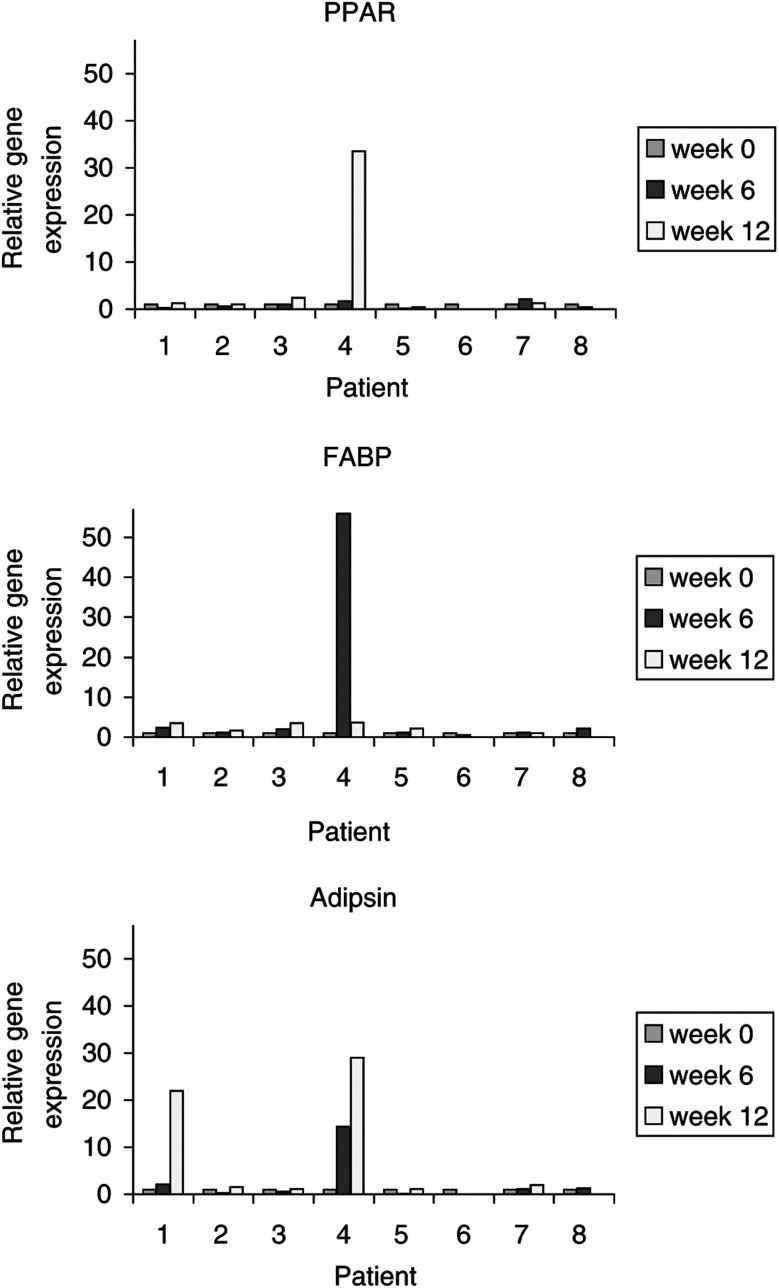
Relative gene expression of PPAR*γ*, fatty acid binding protein and adipsin during treatment with rosiglitazone

Next, we examined the gene expression level of PPAR*γ*, adipsin and fatty acid binding protein, three genes that are specifically linked to adipocytic differentiation. This accurate method would allow a proof of principle of the treatment and a correlation with a potential response. Biopsy material of eight patients could be evaluated. Only one patient, no. 4, had a significant but inconsistent upregulation of all three genes after 6 and/or 12 weeks. This patient had a dedifferentiated liposarcoma and was treated with rosiglitazone for 10 weeks until progression was noted. One other patient, no. 1, had upregulation of only one gene product, adipsin, after 12 weeks of treatment. This patient had a myxoid liposarcoma and continued medication for 6 months before progression was noted. For the six other patients no rise in gene expression level could be found (Figure 2

).

## DISCUSSION

The strategy of activation of PPAR*γ* to induce differentiation or maturation in cells is increasingly receiving attention in cancer treatment during the last few years. It is an attractive model in anticancer therapy, because it has a targeted molecular basis and acts with little side effects, in contrast to chemotherapy. Since adipose tissue has the highest expression of PPAR*γ*, liposarcomas have a theoretical advantage using this treatment as compared to other tumours. Mainly *in vitro* studies offer substantial evidence for the redifferentiation mechanism by stimulating PPAR*γ*. So far one clinical study with three patients with liposarcomas has shown some effect, albeit with a short follow-up period.

In this phase II study, we sought to find further arguments for the efficacy of this treatment for a longer period of time. We collected clinical, histopathological and biological data of nine patients that were treated with rosiglitazone. However, our results were disappointing. The mean progression free survival period was 5.5 months, realising that an uncommon definition of progressive disease was used, namely a 100% tumour volume increase. Morphologically, no convincing sign of redifferentiation could be detected. Stainings of the Ki-67 antigen did not show a significant decrease, mainly because of the very low baseline values in these tumours. An important effort was made to determine the molecular stimulation of PPAR*γ* by measuring the gene products of its activation at the transcriptional level. Since we saw a limited but significant upregulation of gene expression in two patients, we concluded that our methodology was correct and that it was possible to activate PPAR*γ in vivo*. However, we could not find a correlation between this upregulation and a clinical response in our patients.

Several remarks can be made to these results. This is the first clinical trial that uses rosiglitazone as a stimulator of PPAR*γ*, while other clinical trials have used troglitazone. Rosiglitazone has the theoretical advantage of being more potent than troglitazone (Goldstein, 2000). Hepatotoxicity is seldom seen with rosiglitazone in comparison with troglitazone. Nevertheless, we had to withdraw one patient from the trial due to elevated liver transaminases.

By showing a biological effect in two patients, we could prove at the molecular level that this drug indeed activates PPAR*γ in vivo*. Why we did not see a rise of the gene products in the other patients is not clear. A possible explanation could be an unresponsive PPAR*γ* due to an inactivating mutation. Such mutations have been described in sporadic colon carcinomas (Sarraf *et al*, 1999) and thyroid carcinomas (Kroll *et al*, 2000) but, to our knowledge, not in liposarcomas. Other transcriptional errors can of course also be present in these malignant cells.

More important is the fact that the apparent biological activation of PPAR*γ* in these two patients had no clinical or histopathological counterpart. Stimulation of PPAR*γ* has been shown to lead to apoptosis in a colon cancer cell line (Chen *et al*, 2002). However, treatment of patients with metastastic colon cancer with troglitazone is ineffective (Kulke *et al*, 2002). The anticancerous effect of thiazolidinediones has also been shown to be independent from PPAR*γ* activition using PPAR*γ*^−/−^ embryonic stem cells, showing inhibition of translation initiation (Palakurthi *et al*, 2001) as the antitumoral activity. And recently rosiglitazone was proven to inhibit tumour growth and metastasis by inhibiting angiogenesis (Panigrahy *et al*, 2002). These data are in contradiction with the redifferentiation model by the activation of the receptor, but can explain a possible antitumoral effect of this drug.

Human malignancies very rarely harbour mutations of the PPAR*γ* gene (Ikezoe *et al*, 2001, 38/id). This implies that aiming at PPAR*γ* as a specific antitumoral target is not likely to be successful. As for liposarcomas, more knowledge needs to be gained about the fundamental biologic alterations, especially about the translocation that characterises some of the subtypes.
